# Advanced Aboveground Spatial Analysis as Proxy for the Competitive Environment Affecting Sapling Development

**DOI:** 10.3389/fpls.2019.00690

**Published:** 2019-05-28

**Authors:** Peter Annighöfer, Dominik Seidel, Andreas Mölder, Christian Ammer

**Affiliations:** ^1^Silviculture and Forest Ecology of the Temperate Zones, University of Göttingen, Göttingen, Germany; ^2^Department A (Forest Growth), Northwest German Forest Research Institute, Göttingen, Germany

**Keywords:** biomass allocation, tree morphology, competition, light gradient, spatial analysis, growth environment, terrestrial laser scanning, hemispherical photography

## Abstract

Tree saplings are exposed to a competitive growth environment in which resources are limited and the ability to adapt determines general vitality and specific growth performance. In this study we analyzed the aboveground spatial neighborhood of oak [*Quercus petraea* (Matt.) Liebl.] and beech (*Fagus sylvatica* L.) saplings growing in Germany, by using hemispherical photography and terrestrial laser scanning as proxy for the competitive pressure saplings were exposed to. The hemispherical images were used to analyze the light availability and the three-dimensional (3D) point clouds from the laser scanning were used to assess the space and forest structure around the saplings. The aim was to increase the precision with which the biomass allocation, growth, and morphology of the saplings could be predicted by including more detailed information of their environment. The predictive strength of the models was especially increased through direct neighborhood variables (e.g., relative space filling), next to the light availability being the most important predictor variable. The biomass allocation patterns within the more light demanding oak were strongly driven by the space availability around the saplings. Diameter and height growth variables of both species reacted significantly to changes in light availability, and partly also to the neighborhood variables. The leaf morphology [as leaf-area ratio (LAR)] was also driven by light availability and decreased with increasing light availability. However, the branch morphology (as mean branch weight) could not be explained for oak and the model outcome for beech was hard to interpret. The results could show that individuals of the same species perform differently under constant light conditions but differing neighborhoods. Assessing the neighborhood of trees with highly precise measurement devices, like terrestrial laser scanners, proved to be useful. However, the primary response to a dense neighborhood seemed to be coping with a reduction of the lateral light availability aboveground, rather than responding to an increase of competition belowground. The results suggest continuing efforts to increase the precision with which plant environments can be described through innovative and efficient methods, like terrestrial laser scanning.

## Introduction

Every plant competes with other plants or organisms for limited resources. The survival and general plant performance is primarily determined by the amount of resources the individual plant can capture. More specifically, the resource availability influences competitive ability (Funk and Vitousek, 2007), primary and secondary growth (Pretzsch et al., 2017), biomass allocation (McConnaughay and Coleman, 1999; Shipley and Meziane, 2002), and plant morphology (Hutchings and de Kroon, 1994; Kimmins, 2004). For saplings growing in the understory of (temperate) forest ecosystems the amount of light is considered to be among the most important resources (Lambers et al., 2008). Aside of its importance, the availability of light is known to influence different species in various ways, especially when considering light demanding or shade tolerant species (Poorter, 1999; Lödige et al., 2014; Annighöfer et al., 2017), resulting in species-specific traits as reaction to varying levels of light (Petriţan et al., 2009; Schall et al., 2012). Several studies were able to measure the light availability and show its effects on the sapling performance (e.g., Beaudet and Messier, 1998; Williams et al., 1999; Drever and Lertzman, 2001; Claveau et al., 2002; Ammer, 2003; Delagrange et al., 2004; Messier and Nikinmaa, 2016; Annighöfer et al., 2017). Plants in general also compete for other resources above- and belowground, including water, nutrients and growing space (Casper and Jackson, 1997; Leuschner and Ellenberg, 2017). Effects of belowground competition on sapling performance induced by mature trees have been shown by exclusionary experimental setups, e.g., root-trenching experiments (Leuschner et al., 2001; Ammer, 2002; Petriţan et al., 2011), even though still comparably little is known about belowground competition.

In addition to directly measuring the availability of resources and relating it to plant performance, or directly measuring the resource uptake by individual plants (e.g., Ehleringer and Dawson, 1992; Silla and Escudero, 2003), a common approach to explain plant performance is to measure the competitive pressure an individual is exposed to (e.g., Wagner et al., 2009; Seidel et al., 2015). For mature trees, several indices exist to quantify the aboveground competitive pressure an individual might be exposed to at its growth site, among which the Hegyi index (Hegyi, 1974) is a widely applied measure. For saplings, however, many of the competition measures are laborious to derive in the field and rarely used (e.g., Elliot and Vose, 1995). Furthermore, many competition indices are strongly focused on the dimensions and competitive interactions of neighboring full-grown trees. In light-limited surroundings the abundance of shrubs, grasses and herbs can generally be considered to play an insignificant role for the performance of mature trees, even though some studies also show their effect on ecosystem traits (Gebhardt et al., 2014). For smaller saplings however, the abundance and density of shrubs, grass and herbs in their direct neighborhood can be expected to have a more severe impact (Löf, 2000; Coll et al., 2003; Harmer et al., 2005), but quantifying these is complicated. So far, these vegetation layers are usually described in view of their cover, e.g., through Braun-Blanquet (1932) and other visual assessments (Wagner and Radosevich, 1991), or their effect is studied by setting up desired levels of competition through planting and weeding or the use of herbicides (Morris et al., 1990, 1993; Jylhä and Hytönen, 2006). Also, depending on the growth form, some species of these vegetation layers might intercept light, but other species with similar biomass but different growth form might more strongly capture nutrients or water, making the competitive environment of saplings rather complex (Elliot and Vose, 1995).

In this study, we explored a novel approach to quantify the forest structure and neighborhood of saplings through spatial analysis of terrestrial laser scans (TLS). The quantifications derived from the TLS were combined with light measurements derived from a fisheye-lens camera. We expected that a detailed quantification of the individual neighborhood and surrounding forest structure, as addition to the light measurements, would increase the precision with which plant characteristics can be predicted (comp. Wagner et al., 2011). This expectation is based on the assumption that the general availability of resources is not only related to canopy density and thus light availability above each sapling (determined by overstorey density and recorded with the camera), but also linked to the direct neighborhood within the immediate lateral proximity of the saplings and overall indices of forest structure (recorded with TLS). Combining this information should result in a more accurate proxy of the competitive pressure the saplings are exposed to, respectively. To follow-up on this expectation we decided to harvest saplings of a shade-tolerant (*Fagus sylvatica* L.) and a more light-demanding species [*Quercus petraea* (Matt.) Liebl.] growing along light gradients in the understory of two different Central European temperate forests. Since all individuals of both species had regenerated naturally, we expected their growth, morphology, and biomass allocation to be a result of the resources at their growth site which they could capture.

We hypothesized that (1) traits and general performances (in view of growth and allocation) of saplings could be better predicted by not only using light measurements as explanatory variable, but additionally considering information of the saplings’ immediate neighborhood and the surrounding forest structure. Specific hypotheses concerning biomass allocation, growth and sapling morphology were:

(2) Following the “functional equilibrium hypothesis,” above- and belowground biomass is allocated in the direction of the limiting resource (Brouwer, 1963; Shipley and Meziane, 2002), e.g., increasing light availability or decreasing aboveground neighborhood density result in an increase of the belowground root-mass fraction (RMF) (Hofmann and Ammer, 2008).

(3) Diameter growth increases with increasing light availability (Beaudet and Messier, 1998) and decreasing neighborhood density, whereas height growth increases with increasing light availability (Lüpke, 2004; Lüpke and Hauskeller-Bullerjahn, 2004) but also with increasing neighborhood density.

(4) Mean branch weight increases with increasing light availability and decreases with increasing neighborhood density, especially for shade-intolerant species, like beech (Smith, 1994; Ammer, 2003).

(5) The leaf area ratio (LAR) decreases with increasing light availability and is higher under low light intensities for shade-tolerant species (Poorter and Remkes, 1990; Cornelissen et al., 1996).

## Materials and Methods

### Study Area

The saplings were collected from two study locations in Germany. The oak saplings originated from the forestry district “Unterlüß” in Lower Saxony (52°50′ N, 10°16′ O). The stand under investigation is managed as high forest system. Parts of the stand have been naturally regenerated through removing overstorey trees and maintaining regularly distributed seed trees (shelterwood). Retained overstorey trees are mainly comprised of sessile oak (*Q. petraea*) admixed with scattered individuals of Scots pine (*Pinus sylvestris* L.), Norway spruce [*Picea abies* (L.) H. Karst.] and European beech (*F. sylvatica*) trees. The overstorey has an age of about 130–170 years, depending on the species. The Unterlüß study site is situated in the northwest German lowlands at an altitude of approximately 120 m a.s.l. The climate is temperate oceanic, with an average temperature of 8.4°C year^-1^. The long-term annual precipitation lies around 760 mm year^-1^ (Gauer and Aldinger, 2005). The trees are growing on rather nutrient-poor sandy soils, moderately moist (on average sufficient water supply for plant growth) during the growing season, with top soil layers occasionally running dry or water pooling in the deeper soil horizons. The general growth site conditions are considered quite suitable for sessile oak.

The beech saplings were collected in stands of the forestry district “Leinefelde” in Thuringia (10.36° E, 51.32° N). The stand is also a high forest, dominated by beech with admixed single pedunculate oak (*Quercus robur* L.) and sycamore maple (*Acer pseudoplatanus* L.) trees. The age of the overstorey lies around 130–140 years. Following a mast year and partial target diameter harvest, beech regeneration has established. In winter 2013/2014, additionally six gap cuts (three larger, three smaller gaps, range 500–1170 m^2^; Seidel et al., 2015) were added to study the natural succession in these gaps in a long term project (not part of this study). The Leinefelde site is located at 450 m a.s.l. in the central German low mountain ranges. The climate is temperate with a tendency toward subcontinentality. The average annual temperature is 8.2°C year^-1^ and the annual precipitation lies around 680 mm year^-1^ (Gauer and Aldinger, 2005). The trees are growing on a nutrient-rich clayed-silt soil with sufficient water supply, representing very good growth conditions for beech.

### Sapling Collection and Further Field Measurements

Both study sites were divided into systematic grids of 25 × 25 m (Leinfelde) and 18 × 18 m (Unterlüß) with randomized starting points. This grid was chosen to assure covering a variety of light and neighborhood conditions the saplings were growing in. The grid size was derived from the size of the area under regeneration to result in about 50 sampling points. Saplings were collected along the grid points at both sites, by choosing the sapling closest to the grid point, respectively. The only selection criteria were sapling vitality (free of apparent damage) and sapling dimensions (i.e., height and diameter) to assure morphological and age wise comparability of the saplings. If saplings belonged to a regeneration group, the most vital sapling of the group was chosen. Saplings were identified, marked and harvested as whole plants (above- and belowground plant compartments) for further analysis in the laboratory. This resulted in a total of 51 beech saplings in Leinefelde and 44 oak saplings in Unterlüß (Table 1).

**Table 1:** Properties and dimensions of the beech (Fs) and oak (Qp) saplings in the year of harvest.

	Fs	Qp	*p*-value
Number of observations (n)	51	44	
Mean RCD (cm)	18.18 ± 3.36	19.51 ± 6.93	0.23
Mean H (cm)	189.8 ± 25.11	161.46 ± 57.56	<0.05
Mean age (years)	11.88 ± 1.67	6.61 ± 1.85	<0.05

*Presented are the number of saplings harvested (number of observations), their mean root-collar diameter (mean RCD), height (mean H), and counted stem disc age (mean age) with standard deviation (±SD). The *p*-value shows significant differences between Fs and Qp.*

The light environment the saplings were exposed to prior to harvest, was measured at each harvesting location using an automated fisheye lens camera (Solariscope, Behling SOL300, contact: hb.messtechnik@gaia.de). This device calculates measures of light availability (e.g., indirect site factor (ISF), direct site factor, total site factor, openness, gap fraction, and leaf area index) by automatically analyzing hemispherical photographs according to seven threshold values (comp. Pryor, 2010). At each harvesting location one solariscope measurement was conducted at 2 m above ground (comp. Table 1).

**FIGURE 1:**
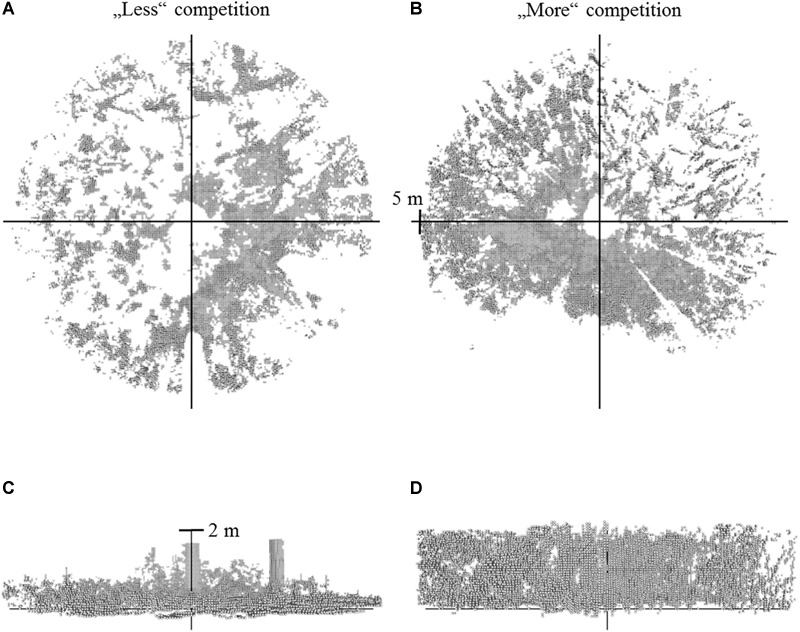
Voxel data (gray) resulting from single scans of two different forest scenes. Left **(A,C)**: situation with less understory vegetation. Right **(B,D)**: situation with more understory vegetation in the vicinity (radius = 5 m) of the sapling (view from top: **A,B**; view from side: **C,D**). Two single stems can be made out in the left view from the side **(C)**, whereby the very dense understory vegetation on the right **(D)** does now allow identifying single objects visually.

Next to the light measurements we assessed the detailed neighborhood conditions and surrounding forest structural conditions of each sapling by taking a TLS at each saplings’ growing position. To ensure capturing the immediate and relevant neighborhood of each sapling each single scan was conducted at 0.8 m above ground, close to 50% of the mean sapling height (comp. Table 1). Scans were captured with the Faro Focus three-dimensional (3D) 120 laser scanner (Faro Technologies Inc., Lake Mary, FL, United States). This terrestrial laser scanner emits 44 Mio laser beams per single scan, which are reflected and received by the instrument, if surrounding objects are hit (comp. Seidel et al., 2015; Ehbrecht et al., 2016). The information stored by the instrument consists of the object’s coordinate in a 3D coordinate system (x, y, z). Since coordinates are dimensionless, they can be converted to voxels (3D pixels) with varying resolution (as presented in Figure 1). Maximum scanning distance of the instrument was 120 m. Resulting 3D point cloud representations of the different forest scenes were processed with FaroScene.

### Sapling Processing and Laboratory Measurements

The harvested saplings were partitioned according to the plant organs leaves, branches, stems and roots. One random fresh-leaf sample of at least 15 leaves was collected from each sapling. These 15 leaves were scanned on a flatbed scanner (Mustek Systems Inc. A3 2400S) and their leaf area was measured with ImageJ (open source; developer: Wayne Rasband). The branches on each sapling were counted. All plant compartments were then dried in a temperature-controlled oven at 70°C to constant weight and weighed (mg). The random leaf samples were dried separately, allowing to use the ratio of leaf area and dry weight to estimate the total leaf area of each sapling. An age estimation and the annual diameter increment of all saplings was measured by extracting a stem disc from each sapling (5 cm above ground), sanding it, and counting and measuring the annual rings, respectively. Year ring widths were measured in two to four (for stem discs strongly deviating from circularity) directions on scanned images (1200 dpi) of the disks, with a precision of 1/100 mm using the software Lignovision (Rinntech version 1.37). Annual height increment was assessed along each stem by measuring the distance between two consecutive annual internodes on the stem surface. Additionally, the root-collar diameter (RCD) was measured 5 cm above ground (where the stem disc was extracted) and the total height (H) of the sapling was measured along the stem axis by measuring its length.

### Data Analysis

#### Analyzing the Growth Environment – Light Environment

Analyzing the light environment was based on the Solariscope measurements. From the resulting seven threshold-based interpretations of the hemispherical photos from each harvesting location, the operator needs to visually choose the one showing the best distinction between sky and non-sky. Two independent operators underwent this procedure for quality assurance, especially for stand conditions which are well represented by more than one threshold value. The quantification of the light environment was based on the ISF. The ISF quantifies the proportion of indirect or diffuse radiation reaching the measurement point.

#### Analyzing the Growth Environment – Forest Structure

**FIGURE 2:**
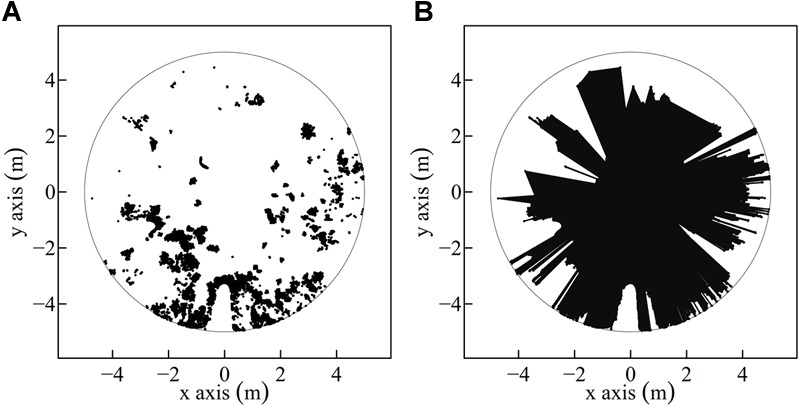
Exemplary understory three-dimensional (3D) point cloud projection onto a two-dimensional (2D) horizontal plane. Transformation of a two-dimensional (2D) point cloud (scatterplot) **(A)** within a radius of 5 m to a polygon **(B)** by connecting all points. The polygon area is colored black **(B)**.

The forest structure was analyzed by calculating published indices based on the full 3D point clouds or parts of the 3D point clouds created through the TLS (single scans). These indices were the effective number of layers (Ehbrecht et al., 2016), the stand structural complexity index (Ehbrecht et al., 2017), and the so called MeanFrac, which is part of the stand structural complexity index presented in Ehbrecht et al. (2017), but used in isolation here. MeanFrac stands for the mean fractal dimension index, based on McGarigal and Marks (1995). It is defined as the arithmetic mean of perimeter to area ratios of vertical point cloud polygons, similar to what can be seen in Figure 2B. A detailed description of its calculation is presented in Ehbrecht et al. (2017).

#### Analyzing the Growth Environment – Neighborhood

The 3D point clouds derived from the single scans were further used to analyze the direct neighborhood of the saplings. We assumed that the immediate neighborhood of a sapling was well represented by all elements of neighboring plants located in a cylindrical selection of the point cloud with a radius of 5 m around the target sapling. For separating the neighborhood information from the light measurements (space vs. light), the cylinder was reduced to a height of 2 m, to only include information on the neighborhood not already recorded by hemispherical photography. These point cloud cylinders were further processed and analyzed applying two different approaches:

The first approach was based on creating voxel-models from the 3D point clouds. The voxel size was 125 cm^3^ (5 cm side length) and voxels served as proxy to calculate a relative space filling value around the individual saplings (Figure 1). An edge length of 5 cm was considered appropriate to maintain the details of the point clouds representing a forest scene, without giving too much weight to single stray points in the original point cloud. Relative filling values were calculated for the full cylinder (radius = 5 m, height = 2 m) and for gradual radius reductions in steps of 1 m by applying the following formula:

(1) $$RF_{i}=\frac{V\_vox\times n\_vox_{i}}{\pi \times R_{i}^{2}\times H}\times 100$$

[with RF_i_ = relative filling of cylinder with radius i; V_vox = volume of voxel (125 cm^3^); n_vox_i_ = number of voxels in cylinder with radius i; R_i_ = radius i of cylinder (with i = 500, 400,…100 cm); H = height of cylinder (200 cm)].

In the second approach, point clouds were not voxeled but each point coordinate was used to span a horizontal polygon, as proxy for the space available around the saplings within the predefined cylinder. Also here resulting polygon areas were calculated for the maximal radius of 5 m and for successive reductions of the radius. To create the polygons, the 3D points within the cylinder were projected onto a two-dimensional (2D) surface, whereas the points below 30 cm height were excluded to reduce the effect of forest ground hits (Figure 2).

#### Analyzing the Saplings

The effects of light availability, forest structure, and neighborhood on biomass allocation (root, stem, branch, and leaves), increment measurements (height and diameter), and sapling morphology (mean branch weight, LAR) were assessed. Mass fractions of each plant organ were calculated in relation to the total plant mass by applying Eq. 2 for each sapling.

(2) $${\text{MF}}_{\text{i}}\text{=}\frac{{\text{BM}}_{\text{i}}}{{\text{BM}}_{\text{T}}}$$

[with MF_i_ = mass fraction of plant organ i (g g^-1^); BM_i_ = biomass of plant organ i (g); BMT = total biomass of sapling (g), with i = root, stem, branch, and leaf].

Next to absolute diameter and height increment measures (comp. above), the increment measures were also converted to relative values (Eq. 3) for each sapling:

(3) $$\text{Rel}\_{\text{inc}}_{\text{i}}\text{=}\frac{{\text{Inc}}_{\text{i}}}{\text{i}}$$

[with Rel_inc_i_ = relative increment of i (1/100); Inc_i_ = increment of i (cm; 1/100 mm), with i = height, diameter (cm; 1/100 mm)].

The morphology was quantified by using the total number of branches to calculate the mean branch weight (Eq. 4) for each sapling:

(4) $$\text{Mean}\_\text{BW=}\frac{\text{B}\_\text{bm}}{\text{B}\_\text{n}}$$

[with Mean_BW = mean branch weight (g n^-1^); B_bm = branch biomass (g); B_n = number of branches].

The morphology was further quantified by calculating the LAR (Eq. 5) for each sapling:

(5) $$\text{LAR=}\frac{\text{LA}}{{\text{BM}}_{\text{T}}}$$

[with LAR = leaf area ratio (cm^2^ g^-1^); LA = total leaf area (cm^2^); BM_T_ = total biomass of sapling (g)].

### Statistical Analysis

The comparisons between groups (e.g., Table 1, between “Beech” and “Oak”) were conducted with parametric and non-parametric tests, depending on the data structure. For data that was normally distributed (Shapiro–Wilk test) and had homogenous variances (Fligner–Killeen test of homogeneity of variances) an analysis of variance model (AOV) was used. For data that was not normally distributed or data that had inhomogeneous variances, a non-parametric Kruskal–Wallis rank sum test was applied.

The relationships between response (y) and explanatory (x) variables were analyzed with multiple-regression techniques. Data was separately analyzed for both species. To reduce the total number of predictor variables and especially to reduce variance inflation through collinearity, single predictors were removed from pairs of predictors with *R*^2^ > 0.7, in reference to Dormann et al. (2013). The remaining explanatory variables were (1) the ISF, as descriptor for the light environment the saplings were exposed to, (2) the relative filling of the cylinder with 5 m radius (RF_5m) and the (3) horizontal polygon area within a radius of 2 m (PA_2m), as descriptors for the direct neighborhood of the saplings; (4) MeanFrac as descriptor for the complexity of the general forest structure of the stands where the saplings were growing.

The multiple regressions as generalized linear models (GLM) were started as full models, including interactions between the light measure (ISF) and the neighborhood variables (RF_5m and PA_2m) as representatives for competitive interactions in view of different resources. To correct for inhomogeneous standard deviations (non-normally distributed regression residuals, visual verification, Shapiro–Wilk test), logarithmic transformations were applied (McDonald, 2014), wherever necessary. The full models were then simplified through backward selection, starting with non-significant interactions and then subsequently removing single variables with highest *p*-values until all remaining variables were significant or significant in interactions, controlled through AOV tables (Crawley, 2013). Models were evaluated through residual standard error (RSE), R^2^ (for models with a single predictor variable) and adjusted R^2^ (as robust measure against overfitted models with more than a single predictor variable). The relative importance of significant explanatory variables in models with more than one explanatory variable was assessed using the proportional marginal decomposition method and metric proposed by Feldman (2005) and recommended by Groemping (2006). For higher order terms (interactions) variable importance was assessed through R^2^ partitioning by averaging over orders according to Lindeman et al. (1980) and also as recommended by Groemping (2006).

Significant interactions were visualized in 3D plots. Selected variables comparing the response of beech and oak to one another were visualized through non-linear Generalized Additive Modeling (GAM) techniques (Hastie and Tibshirani, 1990; Wood, 2006). The GAM visualization was used for comparison because no data transformation was required. The effective degrees of freedom (EDF) were limited to a maximum of 3 (number of knots = 4). However, the amount of smoothing was chosen automatically through generalized cross-validation (Cianelli et al., 2004). The data family was set to Gaussian type with an identity-link function (Wood, 2011). The statistical significance of the GAM models was evaluated by considering the *p*-values tested by an *F*-test.

The significance level for this study was *p* < 0.05. All statistical analyzes, model fitting, and graphs were processed and produced using the free software environment R, version 3.4.0 (R Core Team, 2017).

## Results

The four predictor variables were able to explain 18 out of 20 response variables (unexplained: branch mass fraction, mean branch weight of oak). For these 18 response variables the model quality varied considerably, with adjusted *R*^2^-values ranging from around 0.1 (e.g., stem mass fraction and leaf mass fraction of beech) up to maximum values around 0.5 (absolute diameter increment of oak; absolute and relative diameter increment of beech). A total of 7 out of 18 response variables was explained best by a single variable, which were light availability (ISF, *n* = 4) and relative space filling (RF_5m, *n* = 3). All other models had more than one predictor variable, with their relative importance ranging from 0.03 up to 0.52, respectively. The most abundant significant predictors in all models were the light availability (ISF, *n* = 16, including *n* = 2 significant interactions), followed by the relative space filling (RF_5m, *n* = 11, including *n* = 2 significant interactions). MeanFrac was significant in *n* = 4 models and the polygon area (PA_2m) was significant in *n* = 2 models (comp. Table 2).

**Table 2:** Generalized linear model (GLM) performance table for both species (SP), oak (Qp), and beech (Fs).

SP	Resp	Pred	Est	*p*-value	*R*^2^	Adj. *R*^2^	RSE	Rel. Imp.
Qp	RMF	(Int)	0.3243	0.000	0.40	0.37	0.065	
Qp	RMF	RF_5m	-0.0412	0.001				0.23
Qp	RMF	PA_2m	0.0208	0.003				0.17
Qp	SMF	(Int)	0.1860	0.006	0.31	0.29	0.092	
Qp	SMF	RF_5m	0.0664	0.000				1.00
Qp	LMF	(Int)	0.2030	0.000	0.33	0.28	0.019	
Qp	LMF	ISF	-0.0040	0.010				0.04
Qp	LMF	RF_5m	-0.0340	0.001				0.14
Qp	LMF	ISF:RF_5m	0.0010	0.005				0.15
Qp	BMF	(Int)	0.1606	0.000	0.00	0.00	0.062	n.a.
Qp	LAR	(Int)	48.2485	0.000	0.24	0.18	5.221	
Qp	LAR	ISF	-0.8750	0.042				0.03
Qp	LAR	RF_5m	-7.5537	0.005				0.11
Qp	LAR	ISF:RF_5m	0.2178	0.027				0.10
Qp	Mean_BW (log)	(Int)	0.5716	0.000	0.00	0.00	0.800	n.a.
Qp	D_inc (log)	(Int)	4.1020	0.000	0.50	0.48	0.239	
Qp	D_inc (log)	ISF	0.0241	0.000				0.43
Qp	D_inc (log)	MeanFrac	0.1298	0.015				0.07
Qp	H_inc (log)	(Int)	3.6410	0.000	0.32	0.29	0.456	
Qp	H_inc (log)	ISF	0.0162	0.019				0.11
Qp	H_inc (log)	PA_2m	-0.1545	0.002				0.21
Qp	D_inc_rel (log)	(Int)	-2.9953	0.000	0.22	0.21	0.365	
Qp	D_inc_rel (log)	ISF	0.0184	0.001				1.00
Qp	H_inc_rel (log)	(Int)	-2.7335	0.000	0.20	0.18	0.473	
Qp	H_inc_rel (log)	ISF	0.0220	0.003				1.00
Fs	RMF	(Int)	0.3852	0.000	0.21	0.18	0.038	
Fs	RMF	ISF	0.0016	0.009				0.10
Fs	RMF	MeanFrac	-0.1058	0.007				0.11
Fs	SMF	(Int)	0.5427	0.000	0.08	0.06	0.046	
Fs	SMF	RF_5m	-0.0328	0.042				1.00
Fs	LMF	(Int)	0.1215	0.000	0.13	0.11	0.011	
Fs	LMF	ISF	-0.0004	0.010				1.00
Fs	BMF	(Int)	0.0613	0.111	0.23	0.21	0.031	
Fs	BMF	RF_5m	0.0401	0.000				1.00
Fs	LAR (log)	(Int)	3.8907	0.000	0.46	0.45	0.211	
Fs	LAR (log)	ISF	-0.0203	0.000				1.00
Fs	Mean_BW (log)	(Int)	-1.1926	0.049	0.22	0.18	0.466	
Fs	Mean_BW (log)	ISF	0.0191	0.008				0.11
Fs	Mean_BW (log)	RF_5m	0.4127	0.012				0.10
Fs	D_inc	(Int)	233.4056	0.001	0.58	0.56	43.848	
Fs	D_inc	ISF	5.3324	0.000				0.52
Fs	D_inc	MeanFrac	-118.4924	0.009				0.06
Fs	H_inc	(Int)	-4.5314	0.716	0.23	0.20	9.763	
Fs	H_inc	ISF	0.4246	0.005				0.13
Fs	H_inc	RF_5m	8.6585	0.012				0.10
Fs	D_inc_rel (log)	(Int)	-2.2205	0.000	0.51	0.49	0.236	
Fs	D_inc_rel (log)	ISF	0.0249	0.000				0.46
Fs	D_inc_rel (log)	MeanFrac	-0.5124	0.033				0.05
Fs	H_inc_rel	(Int)	0.0067	0.915	0.23	0.20	0.049	
Fs	H_inc_rel	ISF	0.0024	0.002				0.16
Fs	H_inc_rel	RF_5m	0.0364	0.034				0.07

*Presented are response variables (Resp) and significant predictor variables (Pred) with their estimates (Est), significance level (*p*-value) and relative importance (Rel. Imp.; n.a. = not applicable). Model performance is presented as R^*2*^/adjusted R^*2*^ (Adj. R^*2*^) and residual standard error of the models (RSE). RMF, root-mass fraction; SMF, stem-mass fraction; LMF, leaf-mass fraction; BMF, branch-mass fraction; LAR, leaf-area ratio; Mean_BW, mean branch weight; D_inc, absolute diameter increment; H_inc, absolute height increment; D_inc_rel, relative diameter increment; H_inc_rel, relative height increment; ISF, indirect site factor; RF_5m, relative filling of the cylinder with 5 m radius; PA_2m, horizontal polygon area within a radius of 2 m; MeanFrac, mean fractal dimension index; int, model intercept.*

### Biomass Allocation

**FIGURE 3:**
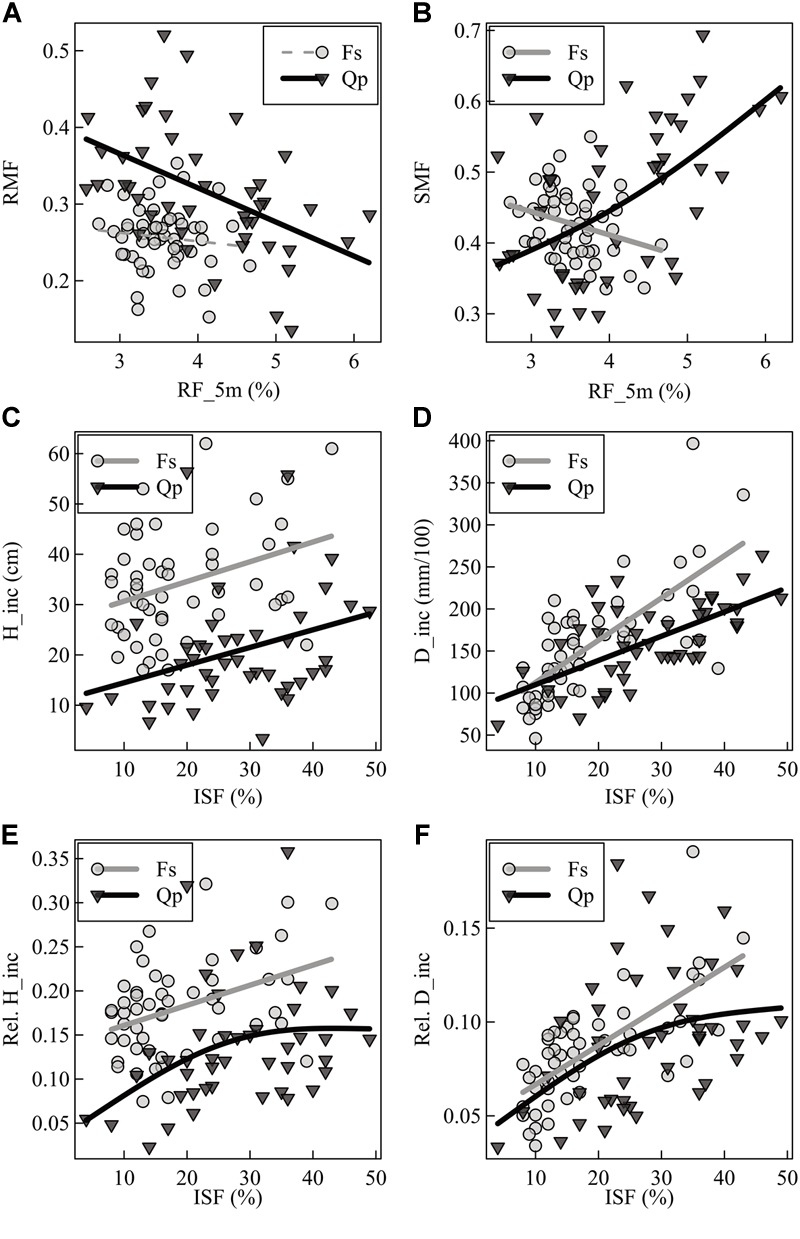
Generalized additive model (GAM) visualization of two selected single predictors (relative space filling = RF_5m; indirect site factor = ISF) of **(A)** root-mass fraction (RMF), **(B)** stem-mass fraction (SMF), **(C)** absolute height increment (H_inc), **(D)** absolute diameter increment (D_inc), **(E)** relative height increment (Rel. H_inc), and **(F)** relative diameter increment (Rel. D_inc) for direct response comparison of beech (Fs) and oak (Qp) saplings. Solid bold lines show significant trends, dotted thin lines show non-significant trends at the level of *p* < 0.05.

**FIGURE 4:**
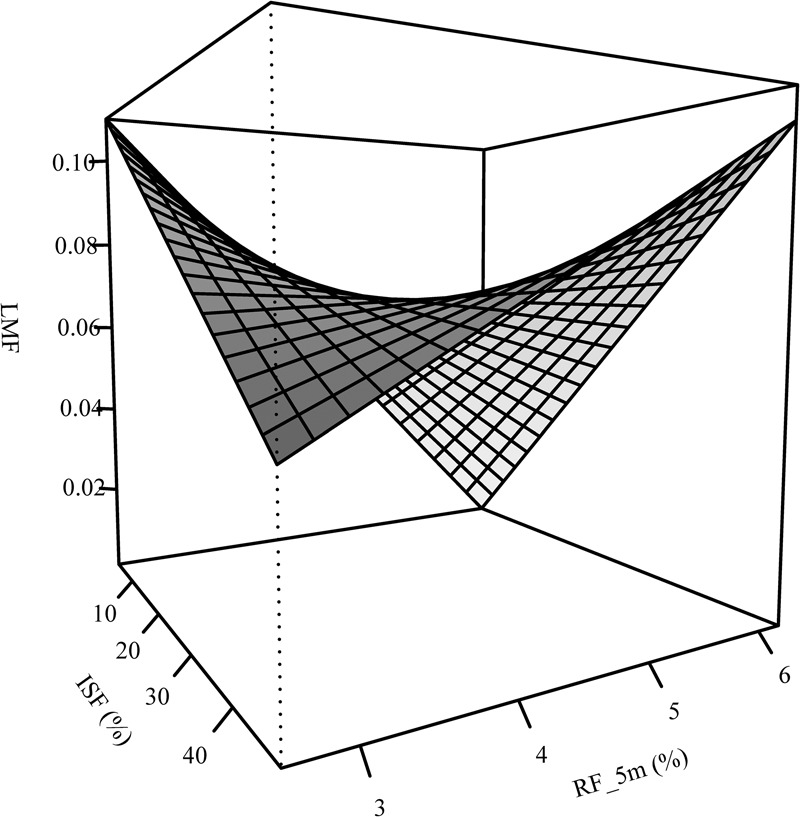
Interaction between light (ISF) and relative filling (RF_5m) as explanatory variables of the leaf-mass fraction (LMF) – analogous to leaf area ratio (LAR) – for the oak saplings.

The RMF of oak was sensitive to neighborhood variables but not to light and forest structure (Table 2). An increase of relative space filling (RF_5m) reduced the RMF, whereas an increase in space (PA_2m) around the sapling increased the RMF. The RMF of the beech saplings did not respond to the neighborhood variables, but only reacted positively to light availability (ISF) and negatively to structural complexity (MeanFrac). The explanatory strength of the model for beech was much lower than for oak (comp. Figure 3A for RF_5m). Stem-mass fraction for oak increased with increasing RF_5m and could nearly not be explained for beech (*R*^2^ = 0.08), even though RF_5m was also the significant predictor here (Figure 3B and Table 2). Leaf-mass fraction generally decreased with increasing ISF, whereas it was part of a significant interaction with RF_5m for oak. Figure 4 shows the interaction, as opposed trends. Under low light availability (ISF) and increasing relative space filling (RF_5m), leaf-mass fraction approaches zero. Under low RF_5m and increasing ISF values leaf-mass fraction also decreases. Under high RF_5m and increasing ISF values leaf-mass fraction increases, as also for high ISF and increasing RF_5m. The branch-mass fraction could not be explained for oak, but increased with increasing RF_5m for beech.

### Increment

The absolute and relative height growth (H_inc and H_inc_rel) for oak and beech saplings increased with increasing light availability (ISF) (Figure 3C,E and Table 2). But absolute height growth was partly more strongly driven by neighborhood. It decreased with increasing polygon area (PA_2m) for oak and increased with increasing relative space filling (RF_5m) for beech (same results for relative height increment of beech). Absolute height increment was higher for beech over the whole light gradient. Absolute and relative diameter increment (D_inc, D_inc_rel) for both species was most strongly driven by light availability and increased with increasing ISF (Figure 3D,F). For beech, also MeanFrac was significant, negatively reacting with increasing diameter growth.

### Morphological Traits

The mean branch weight could not be explained by the considered predictor variables for oak. For beech, it increased with light (ISF) and relative space filling (RF_5m), whereas the explanatory strength was also not high for this model.

The LAR of both species decreased with increasing light availability, whereas this trend was explicit for beech, not so for oak (Table 2). The leaf-area ratio (LAR) model for oak contained an interaction term between ISF and RF_5m again and the explanatory strength of the model was not high (Adj. *R*^2^ = 0.18). The trend of the interaction was analogous to leaf-mass fraction (Figure 4). It suggests that oak LAR was low when light availability was high and space filling with competing vegetation was low. In contrast, LAR increased if either light availability decreased or if space filling increased.

## Discussion and Conclusion

Our study confirmed our major hypothesis that (1) adding explanatory variables, describing the neighborhood of saplings, in addition to light measurements increase the predictive power of tree regeneration trait and performance models. The results showed that more than 50% (11/20) of the models contained more than one predictor variable. Out of all other models with a single predictor variable, light availability was not always the one with the highest explanatory power. Also the relative space filling variable (RF_5m) performed well as single predictor. This finding reflects that plants of the same species perform differently under the same level of light availability, depending on their immediate surrounding (Hofmann and Ammer, 2008). The latter may be determined by intraspecific competitors of the same cohort or by competing vegetation such as grasses or herbs. Such differences in growth environment will inevitable result in different levels of resource depletion (water and nutrients) but also affect other mechanisms that shape plant diversity (morphology), like signaling mechanisms and chemical interactions (comp. Schenk, 2006).

By separating the growth space into a zone above 2 m, in which the light availability was measured, and below 2 m, for which the neighborhood variables were derived, we aimed at getting a more precise proxy of the true competitive pressure a sapling is exposed to. By including the neighborhood we tried to give more weight to the resources other than light, i.e., water and nutrients, by assuming a correlation between above- and belowground competition. However, as in many other temperate forests, light proved to be the dominant driver for sapling performance. On the one side, light is considered to be the decisive factor of plant development (Lambers et al., 2008; Leuschner and Ellenberg, 2017). On the other side, responses to a certain level of light *in situ* always also reflect a cumulated effect of other factors (Beaudet and Messier, 1998) that vary among the microsites with differing canopy openness and light regimes (Collins et al., 1985). For example, root competition by overstorey trees for belowground resources corresponds to low light availability. Disentangling these two drivers would require experimental approaches such as trenching (e.g., Ammer, 2002; Petriţan et al., 2011).

The general predictive strength of the models (considering all response variables together) did not differ greatly between the two species, as a possible consequence of their shade-tolerance levels (Niinemets, 2006), neither by the mean *R*^2^-values of the models, nor by the amount of response variables that could be explained (*n* = 8 for oak; *n* = 10 for beech). When looking at the response variables separately, however, a different picture emerged, especially when considering the biomass allocation based on the growth-environment. Here, allocation toward the different plant organs (root, stem, leaves, not for branches) was better explained for oak than for beech (comp. Figure 3A,B and Table 2). So responses of oak seedlings seem to suggest that they are more sensitive to their immediate surrounding in view of allocation patterns.

In general, the findings for oak and beech confirmed our second hypothesis, namely that biomass is invested in the direction of resource limitation, so that increasing light availability or decreasing neighborhood density would result in an increase of RMF. Both species confirmed the hypothesis differently, however. ISF was not significant for oaks but the neighborhood variables were. The oaks reacted quite sensitive to occupied space around them, so reducing RMF when relative space filling (RF_5m) increased and polygon area (PA_2m) decreased. Considering this response of the oak saplings (decreasing RMF), the primary effect of a dense neighborhood seems to be a reduction of the lateral amount of light the oaks receive. This is supported by the increased stem-mass fraction with increasing relative filling (RF_5m). Our results suggest, that young oaks seem to be less sensitive to low overstorey light levels than to reductions in lateral light availability. In this respect, our investigation goes beyond other studies that have not differentiated between the two directions of light. Accordingly, they reported the more general finding that young oaks are sensitive to light availability (Jarvis, 1964; Ziegenhagen and Kausch, 1995; Lüpke and Hauskeller-Bullerjahn, 2004; Annighöfer et al., 2015; Mölder et al., 2019), However, since aboveground biomass is correlated with belowground biomass (e.g., Jackson et al., 1996; Cairns et al., 1997; Litton et al., 2003), an increase of neighborhood density should also lead to an increase in belowground competition for water and nutrients. This would mean that belowground resources might also become limiting with increasing neighborhood density. Following the “functional equilibrium hypothesis” (Brouwer, 1963; Shipley and Meziane, 2002), an increase of RMF to capture the limiting belowground resources could have also been expected (e.g., Pearson et al., 1984; Comeau and Kimmins, 1989). On the contrary, we could not find indications for this effect of neighborhood density. For a light demanding species, like oak, a kind of allocation-prioritization (hierarchical allocation) might come into play. If above- and belowground resources become limiting, biomass might primarily be allocated to the aboveground compartments. This, of course goes at the expense of root biomass and seems to be independent of a simultaneous limitation of belowground resources. Thus, the need to not lose the connection to light, which might be mortal (comp. Messier et al., 1999) and is referred to as “light-seeking strategy” (Beaudet and Messier, 1998), seems to be of higher priority than to develop the rooting system. However, this strategy may be typical for primarily light-limited settings and may be different in water-limited surroundings (Canham et al., 1996; Poorter and Nagel, 2000). McConnaughay and Coleman (1999) studied annual plants and found that biomass allocation did not strongly react to water availability but only to a light and nutrient gradient, which also allows questioning the equality of resource importance for allocation patterns. Presumably, resource importance also changes during ontogeny (Ammer et al., 2008). Interestingly, beech saplings did not react to the neighborhood variables, but to ISF. Their RMF increased and leaf-mass fraction decreased with increasing ISF, which confirmed the second hypothesis. Also a reduced complexity (MeanFrac) of the forest structure around the beech saplings increased the RMF. Apparently the complexity measure is somewhat linked to the light availability (comp. above). Opposite to the oaks, beech saplings showed decreased stem-mass fraction and increased branch-mass fraction with increasing neighborhood density, which stands quite opposite to other studies and meta-analyzes (e.g., Poorter et al., 2012). However, since the SMF model had a low predictive power the finding should be treated cautiously and may be explained by the fact that the gradient of the neighborhood variables was not as large for beech, compared to oak. Aside of a wider gradient, it is also important to mention that the growth site conditions in terms of water and nutrients were considered good for both species. So even with increasing neighborhood densities, the main driver of competition may have been light, because neither water nor nutrients apparently became as limiting.

The third hypothesis (diameter growth increases with increasing light availability) was partly confirmed for oak and beech. The positive trend between light availability and radial growth increment found here has often been reported in literature (e.g., Beaudet and Messier, 1998; Finzi and Canham, 2000; Chan et al., 2003). Expected effects of the neighborhood variables on diameter growth (diameter growth decreases with increasing neighborhood density) could not be found for both species. Neighborhood effects seem to be negligible and superimposed by the high importance of ISF for diameter growth in both cases. However, relative and absolute height growth of both species showed the expected positive relation to ISF (e.g., Collet et al., 1997; Lüpke and Hauskeller-Bullerjahn, 2004), but also to neighborhood (height growth increases with increasing neighborhood density), eventhough the explanatory strength of the models was not as high as for diameter growth. So since a dense neighborhood increased the height growth for both species (negative PA_2m for oak and positive RF_5m for beech), we can conclude for juvenile trees that a reduction of horizontal space paired with maximal vertical light availability significantly promotes height growth.

Aside of promoting juvenile height growth, a further objective of high densities of juvenile trees in forest management is to reduce their branchiness, which was expressed in the fourth hypothesis. It stated that the mean branch weight increases with light availability and space. This was surprisingly not confirmed for oak (no model could be fitted) and only partly for beech. The mean branch weight increased with ISF but also with increasing RF_5m. Latter contradicts with practical experience and scientific publications or textbooks on silviculture (Mäkinen and Hein, 2006; Röhrig et al., 2006; Weidig et al., 2014) and cannot be explained. However, this finding should not be overrated, since the model quality was also rather low.

Finally, in line with many other studies (Popma and Bongers, 1988; Walters et al., 1993; Lusk, 2002), this study confirmed the negative relationship between LAR and ISF as stated in the fifth hypothesis. It also confirmed that the LAR values and absolute slopes were generally higher for the shade tolerant beech. This is explained by the characteristic of shade tolerant species to increase their light interception rates at low light levels, by growing shade-leaves and generally allocating more biomass to their leaves. The increased leaf-mass fraction in combination with a larger leaf area per unit leaf biomass (shade-leaves) leads to a larger leaf area per unit plant mass (LAR) (Poorter, 2001).

Overall our study could only partly disentangle the effects of overstorey tree density from lateral competition by the local neighborhood. This result may be partly due to methodological shortcomings. Maybe the relevant space defined as neighborhood (here height = 2 m, radius = 5 m) should have been kept flexible, because it presumably differs depending on sapling size. We tried to give more weight to the resources water and nutrients, by assuming a stronger correlation between the availability of these resources and the neighborhood recorded by the TLS approach than between their availability and the light environment. Apparently without actual measurements of resource availability (soil moisture, N-availability) a separation is not really possible. Also, increasing the gradient of growth site conditions, especially toward limited conditions, could result in stronger responses to the neighborhood, as opposed to conditions, in which mainly light availability is limiting. A scanner-based point could cannot efficiently discriminate point qualities yet, i.e., a return from a tree trunk with a high DBH or a small DBH, from a branch, a leaf or a blade of grass is simply a return. Current and future approaches combining the information from TLS and photogrammetry or beams widening the range of spectral wavelengths that can be retrieved aim at overcoming this limitation. A continuously improved analysis of growth environments and neighborhoods will further on increase the quality of models predicting traits and characteristics of plants. This should be further elaborated in future studies.

## Author Contributions

PA conceived and designed the study and wrote the manuscript with support and substantial contributions from DS, AM, and CA. PA collected the data. PA and DS performed the data analysis. CA supervised the findings of this work. All authors commented on and contributed to the final version of the manuscript and approved it.

## Conflict of Interest Statement

CA is currently guest editor of Frontiers in Plant Science hosting the research topic “Woody Plants and Forest Ecosystems in a Complex World – Ecological Interactions and Physiological Functioning Above and Below Ground.” The remaining authors declare that the research was conducted in the absence of any commercial or financial relationships that could be construed as a potential conflict of interest.
